# Opportunity for change: Undergraduate training in family medicine

**DOI:** 10.4102/safp.v62i1.5225

**Published:** 2020-12-11

**Authors:** Olukayode A. Adeleke, Busisiwe Cawe, Parimalaranie Yogeswaran

**Affiliations:** 1 Department of Family Medicine, Faculty of Health Sciences, Walter Sisulu University, Mthatha, South Africa

**Keywords:** medical education, COVID-19, assessment, MBChB, curriculum, family medicine

## Abstract

The coronavirus disease 2019 (COVID-19) pandemic has changed the world as we knew it, and medical education is not an exception. Walter Sisulu University (WSU) has a distributed model of clinical training for the Bachelor of Medicine and Bachelor of Surgery (MBChB) programme. To address the challenges occasioned by the pandemic, the Department of Family Medicine and Rural Health undertook a modification of its MBChB VI programme. The changes aim to ensure the protection of all stakeholders and maintain the integrity of the programme, including the assessment. Changes were made in the delivery of the programme and in the way people interact with one another. Continuous assessment was modified, and the oral portfolio examination was introduced as the summative assessment tool. Although COVID-19 threatened the traditional way of teaching and learning, it however provided us with the opportunity to refocus and reposition our undergraduate medical programme.

## Introduction

When the first confirmed case of the coronavirus disease 2019 (COVID-19) was announced in South Africa on 05 March 2020,^1^ it heralded the beginning of a new era. By 20 March 2020, over 1000 cases were reported in the country, indicating the rapidity of the spread of this infection. The lockdown regulations that were announced in response to the pandemic saw schools and institutions of higher learning closed. Closure of the university and face-to-face teaching and learning plunged both students and academics into an unfamiliar, challenging terrain. The academics had to rethink, innovate and adapt to the new reality of the absence of physical interactions to novel approaches to teaching and assessment practices.

The Walter Sisulu University’s (WSU), Faculty of Health Sciences has a distributed programme for the Bachelor of Medicine and Bachelor of Surgery (MBChB) VI class with sites in East London, Mthatha and Port Elizabeth. When the final-year clinical students were permitted to return for training in May 2020 during level-four lockdown, changes had to be made to the programme. This article highlights some of the changes to the MBChB VI programme by the department of Family Medicine and Rural health at WSU.

## The challenges

Traditionally, medicine is learned by the bedside and not only in the classroom,^2^ and COVID-19 pandemic threatened this practice. With the MBChB VI students’ resumption at the three distributed sites, one of the challenges identified was how to ensure the protection of the patients, facilitators and the students during their expected clinical interactions. This is of particular concern because severe acute respiratory syndrome corona virus 2 (SARS-CoV-2) spreads predominantly via droplets and fomites.^3,4^

When the students left their training sites in mid-March 2020, COVID-19 was a new entity, and the students had no knowledge about the disease. It became apparent that the students needed to have some basic understanding about this disease before their resumption, especially with regard to its transmission, use of personal protective equipment (PPE) and other aspects of infection prevention and control. Hence, the challenge was to ensure that the students obtain relevant knowledge on COVID-19 before returning to clinical training.

Prior to the emergence of the pandemic, it was relatively easy to coordinate the department’s academic programme for the clinical classes across the three sites. Lockdown rules, restriction on movement and gathering and social distancing brought about by the pandemic compelled the department to think of new ways to ensure standardisation of training across sites.

At WSU, traditionally, one of the tools used for continuous assessment for MBChB VI was the Objective Structured Clinical Examination (OSCE), consisting of simulated patients (SPs) and laptop-based stations, amongst others. Continuous assessment takes place at the various sites. However, students from distributed training sites come to the main campus for the final examination, an Individualised Process Assessment (IPA), which is like the traditional ‘long case’. The coronavirus disease 2019 made this model unsuitable, and there was a need to develop different assessment modalities, whilst protecting the integrity of the process.

## Programme adaptation

Changes were made to the programme in response to the above challenges. The changes had to conform to the objectives of the programme whilst maintaining constructive alignment of the curriculum, as defined by Biggs.^5,6^

The goals of these changes are:

- to ensure the safety and protection of the patients, students and healthcare workers;
- to encourage the acquisition of COVID-19-related knowledge, skills and attitude amongst students and facilitators; and
- to protect the integrity of the academic programme.

### Changes to the delivery of the programme

Although the university has a functioning online learning platform, the final-year undergraduate medical programme did not utilise it optimally prior to the pandemic. The department has however been using the e-platform extensively for the Integrated Longitudinal Community clerkship in the MBChB V programme at district hospitals. The coronavirus disease 2019 provided the impetus to extend this to the MBChB VI programme. Online resources, discussion groups and self-practice quizzes were provided for the students. Students and staff were well oriented on the changes to the programme. Interestingly, although COVID-19 necessitated limited physical interaction amongst staff at the three sites, the staff felt more connected during this period because of the regular virtual staff meetings that were conducted.

When the students returned in May 2020, we reduced the length of the contact programme from 5 to 6 weeks to cover for the lost time. The 1-week experiential learning at general practitioner (GP) practices was removed and replaced with an online seminar. We believe the reduction of the time is compensated by a combination of online learning and seminars without compromising the quality of training. Further, experiential learning activities that are based outside the hospital, such as visits to old age homes and hospice home visits, were removed from the programme.

### Changes to the way people interact

It was recognised that students needed to acquire sufficient knowledge, skills and attitudes about COVID-19 essential for their protection from infection and appropriate diagnosis and management of patients. Therefore, the students were required to undertake an online course and submit a certificate of completion to be allowed back to the training sites. The course was developed by the Knowledge Translation Unit at the University of Cape Town.^7^ Appropriate PPE was also made available to the students in all the sites.

On their arrival, we screened and tested the students for SARS-CoV-2, based on the clinical guideline at the time.^8^ The university encouraged the students to adhere to standard precautions at all times. At the hospitals, we ensured the students had appropriate PPE and were excluded from rotating in high-risk areas, such as the COVID wards.

Teachers and facilitators are required to adhere to standard precautions in their daily interactions with students to reduce the risks of infection.^1^ The students were divided into smaller groups, with different groups attending clinical areas and the classrooms at different times. The classrooms were changed to bigger venues to accommodate the mandatory social distancing requirement. Bedside teachings were modified to accommodate a fewer number of students at a time and discussions of clerked patients in a seminar room set-up. At the same time, patients were required to wear cloth masks as required by regulations.^9^

### Changes to the assessments

It is critical to harmoniously integrate the various elements of a curriculum, including the teaching and learning strategies, as well as the assessment processes.^10^ Although there are constant debates about the extent to which assessment rewards or undermines real learning, most educators agree that assessment is an integral part of curriculum.^11^

The continuous assessment before COVID-19 comprised patient presentations, logbook evaluation, three patient studies (write-up), mid-block written test, OSCE and a written paper (modified essay questions). After the recommencement of training, high-risk procedures, especially aerosol-generating procedures such as nebulisation of patients and peak flow rate measurement were removed from the logbook. The OSCE examination, which previously consisted of the use of SPs and laptops (showing clinical images and laboratory results), was modified to avoid exposure of the SP to many students and students touching the same computers. We replaced the stations with SPs with questions based on clinical scenarios. Laptop-based questions were replaced by questions projected on a screen to prevent students from touching the same computer. The written examination included clinical scenarios related to COVID-19 to assess students’ understanding of the disease.

Previously the summative assessment consisted of the IPA (or a ‘long’ case). This was considered unsafe during the pandemic for both the students and patients and was replaced with the oral portfolio examination. Traditionally, the portfolio is used as a formative assessment tool; however, emerging views support an increasing use in summative evaluation.^12,13^ To facilitate a smooth transition to the oral portfolio examination, we trained the students and examiners in the portfolio assessment process. Each student prepared five patient studies of patients seen and managed during the 5-week rotation in the department; these were written and submitted online and were subjected to plagiarism check. At the final examination, the examiners chose one of the five patient studies for discussion. The student was graded independently by two examiners by using a standardised rubric. At the end of the 40-min oral examination, feedback was discussed with the student by using the Pendleton’s model.^14^

## Conclusion

Even though COVID-19 has brought a lot of challenges, causing significant disruption to the way of life, it has also provided a lot of opportunities. It has led to heightened perception and practice of safety, improved communication amongst the decentralised sites and more extensive use of the hitherto underutilised e-learning platform. It has provided us with an opportunity to rethink, refocus and reposition the programme for the undergraduate medical training in the Department of Family Medicine and Rural Health.
